# Systematic Literature Review of Research on the Effectiveness of Art Therapy for Chinese Patients with Depressive Disorder

**DOI:** 10.3390/ijerph22091443

**Published:** 2025-09-17

**Authors:** Guochao Xu, Bo Ram Park, Bo Hyun Kim

**Affiliations:** 1 Department of Fine Arts, Cangzhou Normal University, Cangzhou 061001, China; xuguochao616@gmail.com; 2 Department of Creative Arts Psychotherapy, Jeonju University, Jeonju 55069, Republic of Korea; bbo3897@jj.ac.kr

**Keywords:** Chinese depressive disorder patients, art therapy, systematic literature review, depressive disorder, China, effectiveness

## Abstract

This study aimed to systematically review literature on the effects of art therapy in Chinese patients with depression. The review was conducted in accordance with the guidelines of the preferred reporting items for systematic reviews and meta-analyses. We used four Chinese databases (CNKI, CBM, WF, and VIP) to identify studies and dissertations published in China between January 2008 and December 2023, retrieving 368 studies. We applied the inclusion/exclusion criteria based on the Participant, Intervention, Comparison, Outcome, Time, and Study Design criteria and assessed the risk of bias; 34 studies were included. Studies began in 2008 and their number increased in 2018. They mostly comprised research articles. Intervention targets were most often adolescents (≤19 years old) diagnosed with depressive disorder. Art therapy interventions were conducted and comprised 6–10 group therapy sessions that lasted 60–90 min each. Of the 12 techniques used, painting was the most common. Second, art therapy effectively improved affective, social, cognitive, self, and physical aspects. Given the slow development of art therapy in research and clinical practice, we believe our study makes valuable contributions to its advancement in China.

## 1. Introduction

Mental health problems are common worldwide, with one-eighth of the global population reportedly suffering from mental health disorders [1]. Among mental disorders, depression ranks as one of the most prevalent. Globally, an estimated 3.8% of the population experiences depression, affecting over 280 million people [2]. It has also emerged as a major factor affecting the quality of life in China since 2010, with a lifetime prevalence of 6.8% in 2019 [3]. Approximately 75.9% of patients with depression reportedly exhibit role impairment in at least one domain of family, occupation, interpersonal relationships, or social life on the self-rating depression scale (SDS) [3]. When difficulties due to role impairment become too severe, patients with depression make the extreme choice of committing suicide [4].

Depression treatment mainly comprises medication and psychotherapy [2]. Currently, in China, medication is used to treat depressive disorder [5]. However, it is ineffective for 20–30% of patients with depression owing to various problems, such as recurrence, increased suicide attempts, social dysfunction, and chronicization [6,7]. Hence, medication alone may be ineffective in resolving problems related to a higher level of social adaptation, such as the patient’s learning ability, interpersonal relationships, and interactions. Moreover, long-term medication alone increases the risk of recurrence or impairment if medication compliance decreases, making it imperative to strengthen psychotherapeutic interventions for effective treatment [8].

According to Cuijpers et al.’s meta-analysis that compared the effects of various psychotherapeutic and drug treatments, combined psychotherapy and medication were effective at treating severe depression [9]. Phillips et al. discovered that combined medication and psychotherapy were more effective than medication alone and that psychotherapy improved medication compliance [10]. In particular, psychotherapy that used art exhibited significant effects on the treatment of patients with depression and communication problems, such as helplessness [11]. Art therapy provides a valuable modality for patients to express and alleviate internal psychological problems and difficulties [12]. Crucially, for individuals experiencing depression and exhibiting poor verbal self-expression, art serves as a particularly effective tool for building trust and facilitating communication with the therapist [13]. This approach effectively reduces psychological burden and resistance [14]. Furthermore, the structured working activities inherent in art therapy are recognized for reviving patients’ creativity, ultimately contributing to an improvement in depressive moods [15]. Thus, as a method of nonverbal communication for self-expression through artistic work, art therapy is expected to be a highly effective treatment method for patients with depression.

Although studies have reported various advantages of art therapy for treating depression, they have been perceived to have poor reliability for various reasons, such as subjectivity of the measurement instruments, small sample size, lack of blinding, and unclear or unreported randomization methods [16]. To date, China has been focusing on research on the effects of drug therapy for patients with depression. Therefore, we identified the need for a systematic literature review to analyze and integrate the effects of art therapy on Chinese patients with depression. This study aimed to provide basic data for research and clinical practice via a systematic literature review on the effects of art therapy on Chinese patients with depressive disorder. Based on this objective, the specific research questions were as follows: (1) What trends exist for research on the effects of art therapy in Chinese patients with depression? and (2) What are the effects of art therapy in Chinese patients with depression?

## 2. Research Methods

### 2.1. Systematic Literature Review

A systematic literature review (SLR) is a research method to analyze and present combined research results based on a well-defined central question [17]. Because abundant literature is selected based on search methods and inclusion/exclusion criteria, the data collected are clear and objective [18]. Its strengths include transparency and reproducibility, which can help minimize or overcome issues with previous literature review methods, such as vagueness or researcher bias [19]. The Cochrane group defined strict criteria for SLRs. Processes, such as literature search, risk of bias assessment, and the Preferred Reporting Items for Systematic Reviews and Meta-Analyses (PRISMA) flow diagram, ensure its reliability [20]. With growth in the field of art therapy, related research is also increasing. However, limitations exist in generalizing the reported effects owing to differences in the participants, dependent variables, measurement instruments, intervention programs and techniques, and the media used [21]. Thus, a systematic review of the accumulated research results on art therapy is required.

### 2.2. Procedures

Because this was a systematic literature review, not a human trial (a study using human-derived materials or other studies included in the risk criteria for research), exemption from the review was obtained from the Jeonju University institutional review board on 8 April 2024 before commencing the study. Zheng et al. were the first to implement art therapy interventions and studies to treat Chinese patients with depression [22]. Hence, we selected experimental studies on Chinese patients with depressive disorder published in China between January 2008 and December 2023. Based on the National Evidence-based Healthcare Collaborating Agency (NECA) SLR manual [23] and PRISMA SLR reporting guidelines [24], we selected the research topic and central questions, performed a literature search, selected and classified literature, performed coding, assessed the risk of bias, presented the data analysis and results, and derived our conclusions.

Regarding the entire systematic review process, the researcher (first author) and two independent reviewers (not involved in this study) were consulted at each stage to implement the recommended procedures. The two reviewers, who hold doctoral degrees in art therapy, independently replicated the search and classification procedures. The outcomes from the three parties were then compared. Any discrepancies identified in the results were resolved via discussion with an expert (Prof. Park) and a re-examination process, ensuring the reliability of data identification and selection.

### 2.3. Data Collection

To review the effects of art therapy on Chinese patients with depressive disorder, we set the scope of the literature search via the Participants, Intervention, Comparison, Outcome, Time, and Study Design (PICOTS-SD) criteria. Participants were Chinese patients diagnosed with depressive disorder. We excluded cases reported as depressive moods or conditions, as well as the family and caregivers of patients with depression. The method of intervention chosen was art therapy, which included various techniques, such as thematic painting, free painting, coloring, Chinese painting, and mandala painting. Comparisons were made between experimental groups that received art therapy interventions and control groups that did not. The outcomes were the effects of art therapy on affective, social, cognitive, self, and physical factors in Chinese patients with depressive disorder. Time—the duration of the art therapy journey—was not particularly restricted. The setting—the place, time, frequency, duration, and environment in which art therapy was performed—was not restricted. The study design was randomized, with a nonrandomized control. We excluded single-group studies with no control group, case studies, literature reviews, and meta-analyses. The study included journal articles and theses/dissertations. Conference papers, research reports, or similar publications, as well as duplicate or erroneous records, were excluded. Table 1 lists the inclusion and exclusion criteria for the papers of the literature review.

### 2.4. Data Collection Procedure

We used the China National Knowledge Infrastructure database (CNKI), Chinese Biomedical Literature database (CBM), Wan Fang data (WF), and VIP Chinese Journal Service Platform. The keywords for the literature search were the core concepts of our research topic, “art therapy” and “depressive disorder”. Additionally, clinical experts and psychologists in clinical healthcare in China use the term “painting therapy”. Thus, the final selection of keywords was “art therapy”, “creative arts therapy”, “painting therapy”, and “depressive disorder”. Table 2 lists the database search strategy used in this study.

All documents retrieved from the search were screened according to our inclusion and exclusion criteria. First, we used Note Express Web to exclude duplicate studies. Second, we checked the titles of each study and rejected those with an inconsistent publication type or that diverged from our research topic. Third, based on a review of the titles and abstracts, we excluded studies that did not match our research topic and intervention methods. Fourth, based on a review of the full texts, we excluded studies that did not fit our criteria, such as participants, intervention methods, and study design. Finally, we selected the required literature. To analyze the literature, we examined the authors, year of publication, publication type, study design, and participants’ mean age as the general characteristics. Furthermore, for art therapy intervention-related variables, we examined the intervention time, frequency, and duration, number of participants, treatment techniques and media, dependent variable, and results measurement instruments.

Finally, we used the risk of bias (RoB) and risk of bias assessment tool for nonrandomized Studies (RoBANS) to assess the risk of bias for all the selected studies. For the RoB, seven items were assessed, each classified as “high”, “low”, or “uncertain” risk. For the RoBANS recommended in the NECA SLR manual, six items were assessed, each classified as “high”, “low”, or “uncertain” risk.

## 3. Results

### 3.1. Results of the Literature Search

**Figure 1:**
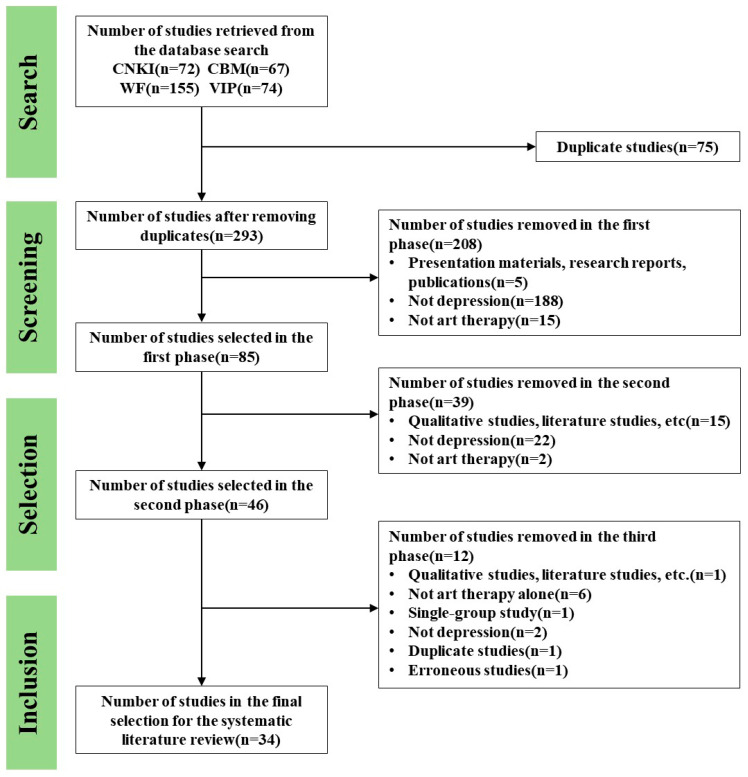
PRISMA flow diagram for the literature selection.

All literature retrieved (*n* = 368) was screened according to our inclusion and exclusion criteria. First, we used Note Express Web to exclude duplicate studies (*n* = 293). Second, the study titles were reviewed, and those that did not match the publication types or differed from our research topic were excluded (*n* = 85). Third, we reviewed the titles and abstracts and excluded any that were inconsistent with our research topic or intervention methods (*n* = 46). Fourth, we reviewed the full texts and selected the final literature, after we excluded those that did not meet our criteria for participants, intervention methods, or study design (*n* = 34). Figure 1 illustrates the literature screen PRISMA flow diagram. Table 3 presents the general characteristics of the included studies.

### 3.2. Risk of Bias Assessment Results

**Figure 2:**
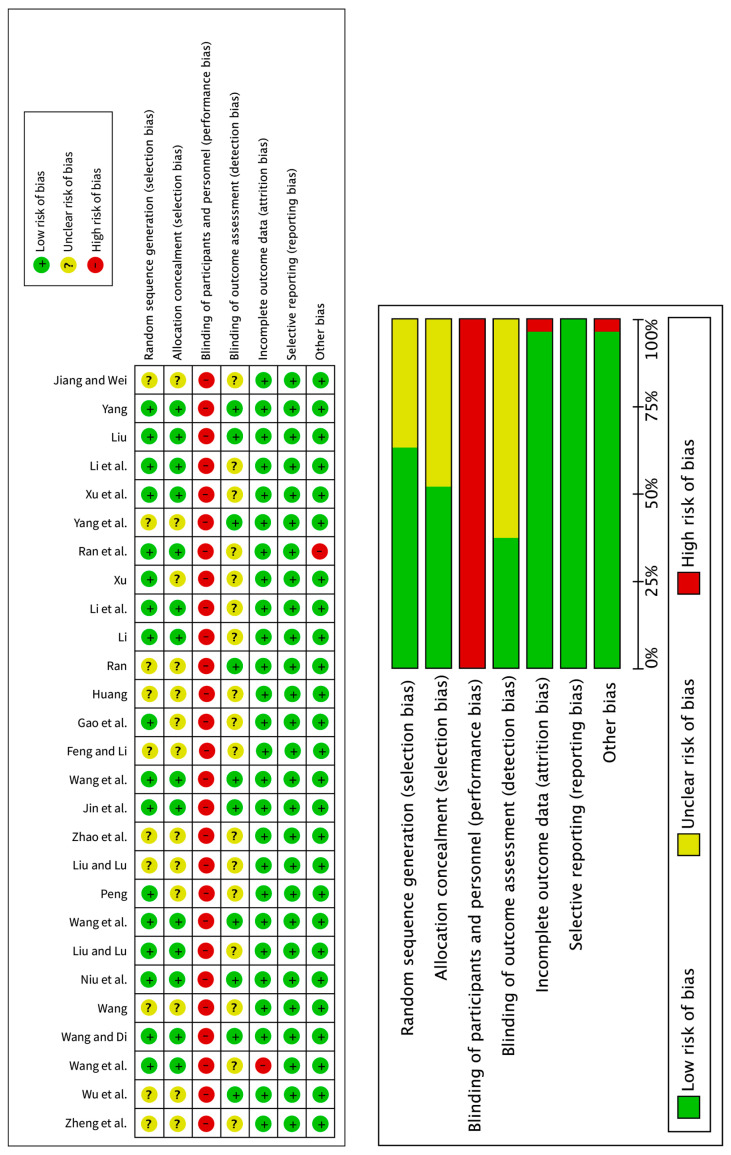
Risk of bias assessment results for the randomized controlled studies [22,25,26,28,29,30,31,32,33,34,35,37,38,39,40,41,42,43,44,45,46,47,48,49,50,51,52,53,54,55].

We used the RoB [20] to assess the risk of bias in 27 randomized controlled studies. Random sequence generation was rated as “uncertain” for 10 studies, as the randomization method was not described in detail. Allocation concealment was rated as “uncertain” for 13 studies owing to missing or insufficient description of random sequence allocation. Blinding of participants and personnel was rated as a “high” risk for all 27 studies, as the nature of the interventions meant that the participants or their caregivers could not be completely blinded during the consent process. Blinding of the outcome assessment was rated as “uncertain” for 17 studies, as there was insufficient information to verify whether blinding had been applied. Incomplete outcome data were rated as a “high” risk for one study, as data values were missing. Selective reporting was rated as a “low” risk for all the studies, as the results were reported in accordance with the study design and criteria. Another bias was rated as a “high” risk for one study, as specific details regarding the art therapy program had not been recorded. Table 4 and Figure 2 present the results of the RoB assessment for the randomized controlled studies.

**Figure 3:**
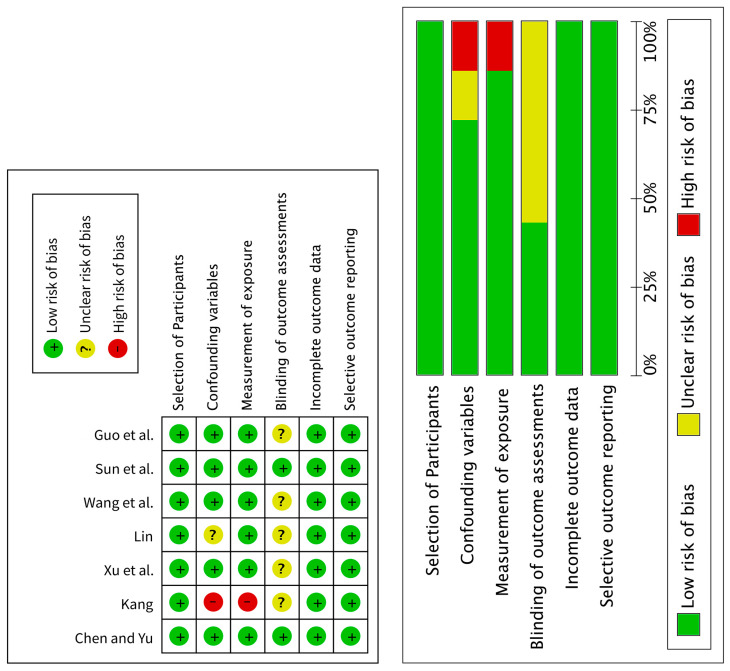
Risk of bias assessment results for the nonrandomized controlled studies [6,8,27,36,47,49,52].

We used RoBANS [20] to assess the risk of bias in seven nonrandomized controlled studies. Confounding variables were rated as “uncertain” for one study, as the randomization method was not described in detail. Measurement of exposure was rated as a “high” risk for one study because specific details about the art therapy program were not recorded. Blinding for outcome assessment was rated as “uncertain” for 4 studies because there was insufficient information to determine whether blinding had been applied to the outcome assessment. Incomplete outcome data were rated as a “low” risk for all seven studies, as the results were reported without missing data in accordance with the study designs and criteria. Table 5 and Figure 3 present the results of the risk of bias assessment for nonrandomized controlled studies.

### 3.3. Trends in Art Therapy Research for Chinese Patients with Depressive Disorder

Of the 34 selected studies, 31 were research articles and three were graduation dissertations. Since the first study on the effects of art therapy for Chinese patients with depression was published in 2008, 0–2 studies were published per year until 2017. Since 2018, 6–7 studies have been published per year, which revealed a trend of increasing research; furthermore, 27 studies have been published in the last six years.

A total of 2374 Chinese patients with depression were included in the studies, with 1188 and 1186 in the experimental and control groups, respectively, among whom 955 were male and 1419 were female. The most common number of participants who received the intervention was 60–69 persons in 11 studies (32.3%), followed by ≤60 persons in 10 studies (29.4%). The most common number of participants in the experimental group was 30–39 persons in 14 studies (41.1%), followed by ≤30 persons and 40–49 persons in nine studies each (26.5%). The participants’ mean age was 33.52 ± 15.36 years, and they were most often adolescents younger than 19 years old (13 studies, 38.3%) or adults aged 30–49 years (11 studies, 32.4%). Looking at participants’ ages, there was a broad range overall, from 10 years old [26] to 80 years old [48]. This demonstrated that art therapy could be implemented without age restrictions.

The intervention time was most often 90 min (11 studies, 32.4%), followed by 60 min (10 studies, 29.4%). The intervention frequency was most often 6–10 sessions (13 studies, 38.2%), followed by 11–15 sessions (seven studies, 20.6%), and the intervention duration was most often four weeks (16 studies, 47.2%). The intervention type was group art therapy in 32 studies (94.1%), although it was not in two studies (5.9%). Thus, we found that group art therapy was more common than individual art therapy, which was consistent with previous findings by Yoon [56].

Twelve techniques were used in art therapy activities: painting, mandala painting, paper crafts, origami, sticking, knitting, collage, clay, calligraphy, Chinese painting, drawing, and coloring. Of these, the most commonly used activity was painting (23 studies, 46.9%), followed by mandala painting (seven studies, 14.4%). Because art therapy is a discipline that originated overseas, exploring the differences between Eastern and Western cultures and integrating them suitably into Chinese culture will contribute to the development of art therapy in China [57]. Therefore, further studies that utilize art therapy techniques with uniquely Chinese characteristics, such as calligraphy, Chinese painting, and folk arts, are required [27,58,59]. Table 6 presents the trends in art therapy research for Chinese patients with depressive disorder.

### 3.4. Effects of Art Therapy Interventions for Chinese Patients with Depressive Disorder

Almost all 31 studies investigating emotional factors reported beneficial effects. Depression represented the most extensively studied subject within this domain, with 27 studies demonstrating statistically significant improvements in depressive symptoms following art therapy interventions. However, Wu et al. reported nonsignificant changes (*p* > 0.05) specifically in somatic symptom factors associated with depression—namely weight fluctuation, diurnal variation, and sleep disturbance—despite significant improvements in other assessed factors [55]. Similarly, Ran et al. found no significant change (*p* > 0.05) in the generalized anxiety factor, indicative of core anxiety symptoms [32]. These findings suggest specific limitations in the therapeutic scope of art therapy, meriting further investigation. Critically, and as highlighted previously, the variables of alexithymia, emotional regulation, and subjective well-being (happiness) were each analyzed in only a single study within this review. Consequently, future research should prioritize examining the effects of art therapy on these specific variables.

Majority of the 12 studies examining social factors reported positive effects. For instance, Xu et al. observed a significant improvement in social functioning following a 4-week group therapy intervention (*p* < 0.001) [30], and Xu emphasized the development of social skills among adolescent patients (*p* < 0.01) [33]. Interestingly, Wang and Di found no significant difference concerning personal hygiene [53], suggesting that the therapy’s effects may be more targeted, focusing primarily on interpersonal relationships rather than daily self-care habits.

All 10 studies examining cognitive factors reported positive effects. For instance, Jiang and Wei found significant improvements in cognitive function following a 4-week group therapy intervention (*p* < 0.001) [25]. Guo et al. highlighted improvements in rumination among female patients (*p* < 0.005) [6]. Peng reported enhancements in patients’ verbal communication skills, manual dexterity, and mental state [45]. These findings collectively suggest that the therapy yields positive effects across multiple facets of social functioning.

All 9 studies examining self-related factors reported positive effects. Yang found a significant increase in self-esteem levels following a 4-week group therapy intervention (*p* = 0.002). Interestingly, a one-month follow-up assessment revealed no significant difference in self-esteem levels between the experimental and control groups. This may be associated with the discontinuation of mandala drawing practice by the experimental group participants post-intervention [26]. These findings suggest that while the therapy demonstrates positive effects on self-related factors, the stability and sustainability of these improvements warrant further investigation.

Finally, among the 5 studies examining physical indicators, 3 reported positive effects. Xu et al. observed significant improvements in medication side effects (e.g., nausea, sleep disturbances, fatigue, and diarrhea) after 4 weeks of art therapy (*p* < 0.05) [47]. Notably, Niu et al. and Ran et al. found no significant difference in the incidence of adverse reactions compared to the control group (*p* > 0.05) [32,50]. However, the therapy demonstrated significant improvements in fatigue [27] and motor function [31]. This suggests that the therapy’s effects on physical indicators are more focused on alleviating fatigue and enhancing motor function, rather than mitigating medication side effects. Table 7 presents the effects of art therapy interventions for Chinese patients with depressive disorder.

## 4. Discussion

### 4.1. Trends in Art Therapy Research for Chinese Patients with Depressive Disorder

Although research on the effects of art therapy for Chinese patients with depressive disorder started relatively late, an increasing trend for research has been observed since 2018, with 27 studies published in the last six years. This increase is not unrelated to the phenomenon of high stress among modern Chinese individuals causing psychological, cognitive, and behavioral impairment and related diseases [60]. This has led to increased awareness and interest in art therapy in China; furthermore, art therapy interventions and research on the treatment of Chinese patients with depression has begun in earnest [61].

Another significant trend pertained to participant gender. Sixty percent of participants were female, which may reflect the significantly higher prevalence of depressive disorders among women compared to men. Interestingly, four studies [6,31,34,44] specifically focused on female patients, whereas none exclusively investigated male patients, highlighting a need for further research in this direction. In contrast to gender, the age range in our sample was broad, spanning from 10 years [61] to 80 years [48]. The mean age distribution was predominantly concentrated among adolescents (38.3%) and middle-aged patients (32.4%). This can be related to the fact that the average age group of Chinese depression patients is 20–30 years [7]. The number of participants in the experimental group was most commonly 30–39 persons (14 studies, 41.1%). The sample size is important in experimental studies as it affects the therapeutic effects and study outcomes [62]. However, discussions on the appropriate sample size for art therapy studies in China are lacking. This was consistent with the results of Lee [63] and Liu [28]. We anticipate that with more active research and discussions on the appropriate sample size for experimental studies on art therapy in China, investigating its optimal effects will be possible.

Intervention session length ranged from 30 to 120 min. Overall, the intervention duration per session was most commonly 90 (32.4%) or 60 min (29.4%) long, the frequency was 6–10 (38.2%) or 11–15 sessions (20.6%), and the duration of the therapy journey was four weeks (47.2%). Interestingly, Wang et al. and Zheng et al. implemented the longest session length at 120 min [22,54]. By contrast, Wu et al. conducted the lengthiest protocol overall, spanning 24 weeks with a total of 168 sessions—representing the longest duration and highest number of sessions among all studies [55]. Such an extensive number of sessions and prolonged duration is highly unusual in art therapy practice. This anomaly may be attributable to the nascent, exploratory stage of art therapy research in China at the time these studies were conducted. The effects of art therapy were strongest when the intervention time per session was 60–90 min [64], and the most common intervention frequency was 11–15 sessions [56,65]. In art therapy, forming a therapeutic relationship and conducting art activities is difficult if the number of sessions is too short. Conversely, if the number is too long, it can have negative effects on the outcomes owing to drop-outs and uncooperative group members [66].

Group art therapy was the most prevalent intervention format (32 studies, 94.1%). In particular, descriptions of the structured intervention protocols within group therapy sessions were often inadequate. For instance, Ran et al. (2021) provided no details regarding the organization of collaborative painting activities or the specific painting protocols employed [32]. Similarly, Kang omitted essential information concerning the specific artistic content and themes utilized in the intervention [49]. Nevertheless, these studies were included because art therapy in China was still in a nascent stage of development and professionalization at the time of their execution, and the general description of the interventions met our inclusion criteria. In particular, given the nature of patients with depression, designing appropriate group art therapy could be directly related to the treatment effects. In group art therapy, the process of sharing works provides opportunities for communication with others, with encouragement and support reducing fear of social activities. Therefore, art therapy helps to improve recovery and social function in patients with depression and promotes participation in and return to society [67].

Various techniques have been used in art therapy interventions for Chinese patients with depression, and these have produced treatment effects. Painting was the most easily accessible, allowed for personal emotions to be expressed in a relatively short time [68], and the most commonly used technique (46.9% of studies). Because art therapy is a discipline that started overseas, using techniques that reflect uniquely Chinese characteristics, such as calligraphy, Chinese painting, and folk art, could contribute to its advancement in China [27,58,59,69].

### 4.2. Effects of Art Therapy Interventions for Chinese Patients with Depressive Disorder

Of the 31 studies, most revealed an effect of art therapy on emotional outcomes in Chinese patients with depression. This supported the claim by Liu and Lu that art provided routes for emotional expression and opportunities for change [70]. Sun et al. reported that the process of voluntary art creation helped reflection through rumination and that the value of art therapy lay in the sharing of this experience [27]. Thus, art therapy is effective in reducing levels of depression and anxiety by allowing patients to feel social support through the experience of expressing psychological difficulties and emotions and close listening and empathy.

Twelve studies revealed an effect of art therapy on social factors in Chinese patients with depression. Even if patients exhibited alleviation of depressive symptoms after medication and psychotherapy, social adaptation and complete recovery of social skills were usually more challenging [71]. Wang and Di found that group art therapy helped improve social communication abilities in patients with depression [53]. Sharing and communication of art activities and works encouraged patients to participate in the group [72]. The process of positive interactions, conflicts, and conflict resolution in a group teaches the patient to understand and control themselves and provides opportunities to practice this in daily living [73]. Thus, art therapy helps social adaptation and functional improvement and increases the quality of life. Therefore, patients can resolve problems and difficulties in their daily living, academic work, family, or interpersonal relationships [74].

Most of the 10 studies revealed an effect of art therapy on cognitive factors in Chinese patients with depression. Neurologists Frith and Law discovered that art could stimulate brain regions related to object identification [75]. When the patient decided what images to express and where to express them, the temporal and parietal lobes became active, respectively. While completing artwork, patients experience an emotional response, and complex interactions arise in the brain in the process of expressing these experiences verbally. Therefore, art therapy helps correct patients’ attention, abstract thinking, verbal expression, and imbalanced or illogical thinking.

Most of the nine studies revealed an effect of art therapy on self-related factors in Chinese patients with depression. Patients’ internal negative emotions were alleviated by art therapy, which also improved self-esteem and self-confidence. Moreover, patients were seen actively interacting with others while participating in group activities. This was consistent with previous studies that reported art therapy helped stabilize patients, improve negative emotions, and also increase self-awareness [32], self-appraisal [25], and self-esteem [37].

Of the five studies, most revealed an effect of art therapy on physical factors in Chinese patients with depression. Furthermore, three studies investigated the effects on adverse responses to medication and reported that art therapy was effective for somatization symptoms caused by psychological difficulties. However, no effect was observed on adverse responses to medications. Meanwhile, another study reported that art therapy was effective for fatigue and upper limb structure/motor function, which was similar to Sun et al.’s report that artistic processes were enjoyable and reduced physical and mental fatigue [27]. This was also consistent with Yang et al.’s study, which reported that art therapy aided voluntary fine motor activity, stimulated the cerebral cortex, and activated neural pathways, which helped restore and improve upper limb function in patients diagnosed with stroke-related depression through the experience of reconstructing normal motor patterns [31].

In summary, art therapy demonstrates beneficial effects across emotional, social, cognitive, self-related, and physical domains for individuals with depression in China. It should be noted that due to the inherent requirement for active participant engagement in art-making, true blinding is unattainable in art therapy interventions. This limitation substantially compromises the internal validity and reliability of findings. To address this methodological constraint, we recommend employing active control conditions (e.g., music therapy, dance therapy, or board games) or incorporating objective outcome measures such as physiological biomarkers and behavioral observations. Furthermore, deviations from standardized protocols may have occurred owing to nonspecialized experimental settings, variability in practitioner qualifications, and suboptimal instrumentation selection, potentially confounding observed outcomes. Future research should prioritize rigorous standardization of experimental protocols to enhance methodological robustness. Finally, while preliminary evidence suggests improvements in several outcome variables, the limited number of studies necessitates further empirical validation to establish the efficacy and generalizability of these effects.

## 5. Conclusions

We retrieved 34 studies on the impact of art therapy for depressed patients published in China between January 2008 and December 2023, analyzing study design, methodology, intervention procedures and techniques, and dependent variable categories. The findings demonstrate that an increasing number of studies in China are examining the effectiveness of art therapy for depression, with the majority reporting positive outcomes. However, our systematic review also revealed that the methodological quality of these studies—particularly owing to deficiencies in blinding implementation—is alarmingly low. Therefore, our research not only summarizes the apparent efficacy but also underscores the critical methodological weaknesses and provides specific recommendations for designing future studies with enhanced rigor and reliability. Furthermore, our study highlights a key methodological paradox: while blinding is inherently unattainable in art therapy, this very limitation necessitates a fundamental reconsideration of future research design. We propose alternative methodological approaches, such as employing active control groups (e.g., music listening, board games) or incorporating objective outcome measures (e.g., physiological biomarkers, behavioral observations). Subsequent research should enhance validity and feasibility by further improving methodological rigor within experimental designs.

Our study had several limitations. First, the 34 studies included both randomized and nonrandomized controlled trials. Owing to the nature of art therapy, qualitative studies were more common; however, difficulties arose in selecting and analyzing them for this systematic review, leading to their exclusion. This review was limited to Chinese databases, which restricts the international generalizability of the findings to some extent. Additionally, owing to language barriers, relevant studies conducted in China but published in English may have been overlooked. Future studies should expand the scope of the review to derive more diverse results.

Second, substantial heterogeneity was observed across the included studies, stemming from significant variations in the duration of art therapy interventions and the specific intervention protocols employed, coupled with inconsistent outcome measures. This heterogeneity precluded a formal meta-analysis. Regarding treatment effects on dependent variables, our analysis was limited to determining the presence or absence of an effect; statistical quantification of the precise effect sizes was not feasible. Future research should focus on increasing the number of studies in this domain and incorporating more standardized protocols to address these methodological gaps.

Third, only 2 of the 34 studies included retests four weeks after the conclusion of the intervention. Verification of the persistence of and generalization of the effects of art therapy presented challenges. Future studies should perform retests after the conclusion of the intervention to verify the persistence of the effects.

Fourth, in some studies, patients’ demographic characteristics or the intervention process were not clearly described, which hindered analysis. Therefore, future studies should provide readers with the required basic data by specifically and accurately recording information such as demographic characteristics, intervention process, and the study design.

## Figures and Tables

**Table 1:** Inclusion and exclusion criteria for papers subject to literature review.

Category	Inclusion Criteria	Exclusion Criteria
Year of publication	1 January 2008—31 December 2023	All literature outside the inclusion period
Type of publication	Academic journal articles (published in China) Thesis (published in China)	Conference Papers Reportable papers or publications Seminar reviews or Supplementary Materials
Duplicate literature	Academic journal articles Recent years first	Duplicate publication of academic journal articles Thesis with the same content as the journal article
Subject of mediation	Depression patients Chinese	Patients who have not been diagnosed with depression Non-Chinese Family members or caregivers of patients with depression
Arbitration measures	Art therapy	Art therapy and other art therapies (music, dance, psychodrama, etc.)
Implementer	No special restrictions	-
Research design	Randomized controlled experiment Nonrandomized controlled experiment	Qualitative research (case study) Literature research (trend analysis, meta-analysis, etc.) Control group or individual study
Control group	Same period as the experimental group Observation and measurement without participating in art therapy	When the reference period is unclear or different from the experimental group Asynchronous observation and measurement
Experimental period	No special restrictions	-
Place of experiment	No special restrictions	-

**Table 2:** Database search strategy for this study.

Database	Search Formula
CNKI	(Subject: Painting Therapy) OR (Subject: Arts Therapy) OR (Subject: Art Therapy) AND (Subject: Depression + Depressed Patients); Restrictions: January 2007 to October 2023
CBM	“Painting Therapy” [core field] OR “Arts Therapy” [core field] OR “= Art Therapy” [core field] AND “Depression” [core field]; Restrictions: 2007–2023; Case reports; Clinical experiment; Randomized controlled experiment;
WF	Subject: (“Painting Therapy”) or Subject: (“Arts Therapy”) and Subject: (Depression); Restriction: 2007–2023;
VIP	(Arbitrary field = Painting Therapy OR arbitrary field = Arts Therapy) OR arbitrary field = Art Therapy) AND arbitrary field = Depression); Restriction: 2007–2023;

Core fields consist of three search items: title, keywords, and subject terms; arbitrary fields = subject.

**Table 3:** General characteristics of the included studies.

ID	Author (Year)	Publication Type	N (EG/CG)	Gender (Male/Female)	Age (M ± SD)	Intervention Frequency	Activity Type	Activity Techniques	Measurement Instruments	Measurement Outcomes (*p*)
1	Jiang and Wei [25] **	Research article	N = 100 (EG = 50/CG = 50)	EG: 7/43 CG: 8/42	EG: 16.20 ± 1.40 CG: 15.84 ± 1.35	4 weeks, 2 times/week, 90 min/each	G + M	Painting	PANAS MCCB SES	*p* < 0.001 *p* < 0.001 *p* < 0.001
2	Yang [26] **	Research article	N = 46 (EG = 23/CG = 23)	EG: 6/17 CG: 5/18	EG: 15.52 ± 0.387 CG: 14.96 ± 0.424	4 weeks, 2 times/week, 40–50 min/each	P + M	Painting	SDS SAS RSS SES	*p* < 0.001 *p* = 0.009 *p* < 0.005 *p* = 0.002
3	Sun et al. [27] **	Research article	N = 93 (EG = 48/CG = 45)	EG: 31/17 CG: 29/16	EG: 62.51 ± 5.39 CG: 63.47 ± 5.22	Total 5 times, 30–60 min/each	G + M	Painting Chinese painting	SDS SAS MoCA FSS	*p* < 0.01 *p* < 0.005 *p* < 0.01 *p* < 0.05
4	Guo et al. [6] **	Research article	N = 82 (EG = 42/CG = 40)	EG: 0/42 CG: 0/40	EG: 46.07 ± 6.75 CG: 45.92 ± 6.71	8 weeks, 2 times/week, 90 min/each	G + M	Painting	HAMD ERQ RSS CFQ	*p* < 0.05 *p* < 0.05 *p* < 0.05 *p* < 0.05
5	Liu [28] **	Dissertation	N = 66 (EG = 33/CG = 33)	EG: 5/28 CG: 5/28	EG: 14.55 ± 1.716 CG: 14.76 ± 1.601	8 weeks, 1 time/week, 60 min/each	G + M	Painting	HAMD TAS-20 SES	*p* < 0.001 *p* < 0.001 *p* < 0.001
6	Li et al. [29] *	Research article	N = 156 (EG = 78/CG = 78)	EG: 42/36 CG: 46/32	EG: 38.9 ± 6.3 CG: 40.2 ± 7.2	8 weeks, 3 times/week, 60 min/each	G + M	Mandala painting Painting	HAMD HAMA NOSIE	*p* < 0.001 *p* < 0.001 *p* < 0.001
7	Xu et al. [30] **	Research article	N = 80 (EG = 40/CG = 40)	EG: 18/22 CG: 15/25	EG: 42.30 ± 15.76 CG: 41.15 ± 16.94	4 weeks, 2 times/week, 60 min/each	G + M	Mandala painting	SDS SAS SDSS RESE HHI	*p* < 0.001 *p* < 0.001 *p* < 0.001 *p* < 0.001 *p* < 0.001
8	Yang et al. [31] **	Research article	N = 58 (EG = 29/CG = 29)	EG: 15/14 CG: 14/15	EG: 54.41 ± 7.61 CG: 53.32 ± 7.82	4 weeks, 5 times/week, 30 min/each	G + M	Paper crafts Sticking Knitting	HAMD MBI FMAS	*p* < 0.05 *p* < 0.05 *p* < 0.05
9	Ran et al. [32] *	Research article	N = 42 (EG = 22/CG = 20)	EG: 11/11 CG: 10/10	EG: 13.38 ± 1.17 CG: 13.36 ± 1.27	8 weeks, 1 time/week, 60 min/each	G + M	Not recorded	DSRSC PHCSS TESS	*p* < 0.005 * *p* = 0.001 ** *p* = 0.87
10	Wang et al. [8] **	Research article	N = 98 (EG = 49/CG = 49)	EG: 21/28 CG: 23/26	EG: 18.67 ± 1.34 CG: 18.39 ± 1.16	4 weeks, 5 times/week, 90 min/each	G + M	Painting	BDI BAI SDSS	*p* < 0.001 *p* < 0.001 *p* < 0.05
11	Xu [33] **	Research article	N = 96 (EG = 48/CG = 48)	EG: 27/21 CG: 26/22	EG: 18.11 ± 1.55 CG: 18.03 ± 1.56	4 weeks, 2 times/week, 80 min/each	G + M	Painting	HAMD HAM NOSIE	*p* < 0.001 *p* < 0.001 *p* < 0.05
12	Li et al. [34] **	Research article	N = 80 (EG = 40/CG = 40)	EG: 0/40 CG: 0/40	EG: 28.56 ± 5.72 CG: 27.79 ± 5.14	Total 8 weeks,	G + M	Mandala painting	HAMD SAS GWB	*p* < 0.05 *p* < 0.05 *p* < 0.001
13	Li [35] *	Research article	N = 86 (EG = 43/CG = 43)	EG: 23/20 CG: 22/21	EG: 16.48 ± 2.16 CG: 16.26 ± 2.02	24 weeks, 1 time/week, 50 min/each	G + M	Painting	HAMD GQOLI	*p* < 0.001 *p* < 0.05
14	Lin [36] *	Research article	N = 40 (EG = 20/CG = 20)	EG: 10/10 CG: 10/10	EG: 15.66 ± 1.55 CG: 16.55 ± 1.65	24 weeks, 1 time/week, 50 min/each	G + M	Painting	HAMD	*p* < 0.05
15	Ran [37] *	Dissertation	N = 34 (EG = 17/CG = 17)	EG: 9/8 CG: 7/10	EG: 12.58 ± 1.17 CG: 12.58 ± 1.41	8 weeks, 1 time/week, 90 min/each	G + M	Painting	DSRSC SCARED WCST PHCSS	*p* < 0.001 * *p* < 0.001 ** *p* = 0.08 *p* < 0.001
16	Huang [38] *	Research article	N = 80 (EG = 40/CG = 40)	EG: 22/18 CG: 21/19	EG: 16–21 CG: 17–21	12 weeks, 1 time/week, 30–60 min/each	G + M	Painting	SDS	*p* < 0.05
17	Gao et al. [39] **	Research article	N = 78 (EG = 39/CG = 39)	EG: 16/23 CG: 14/25	EG: 39.00 ± 12.91 CG: 45.00 ± 16.57	4 weeks, 3 times/week, 60 min/each	G + M	Mandala painting Painting	HAMD HAMA NOSIE	*p* < 0.001 *p* < 0.001 * *p* < 0.001
18	Feng and Li [40] *	Research article	N = 90 (EG = 45/CG = 45)	EG: 24/21 CG: 23/22	EG: 16.10 ± 4.1 CG: 16.30 ± 4.1	4 weeks, 2 times/week, 90 min/each	G + M	Painting	PANAS MCCB SES	*p* < 0.001 *p* < 0.001 *p* < 0.001
19	Wang et al. [41] *	Research article	N = 53 (EG = 25/CG = 28)	EG: 12/13 CG: 12/16	EG: 17.96 ± 3.71 CG: 18.21 ± 2.83	4 weeks, 2 times/week, 90 min/each	G + M	Painting Clay Mandala painting	PANAS WCST	*p* < 0.05 ** *p* = 0.08
20	Jin et al. [42] *	Research article	N = 71 (EG = 36/CG = 35)	EG: 16/20 CG: 14/21	EG: 34.58 ± 8.81 CG: 32.06 ± 8.46	4 weeks, 3 times/week, 30 min/each	G + M	Coloring	HAMD HAMA NOSIE	*p* = 0.034 *p* < 0.001 NOSIE
21	Zhao et al. [43] *	Research article	N = 100 (EG = 50/CG = 50)	48/52	All: 20.30 ± 4.60	12 weeks, 1 time/week, 40 min/each	G + M	Painting Collage	SDS SAS SDSS WHOQOL	*p* < 0.001 *p* < 0.001 *p* < 0.001 * *p* < 0.001
22	Liu and Lu [44] **	Research article	N = 62 (EG = 30/CG = 32)	EG: 0/30 CG: 0/32	EG: 32.77 ± 10.15 CG: 33.44 ± 13.97	4 weeks, 2 times/week, 90 min/each	G + M	Painting	SDS PANAS	*p* < 0.05 *p* < 0.001
23	Peng [45] *	Research article	N = 60 (EG = 30/CG = 30)	EG: 11/19 CG: 10/20	EG: 51.1 ± 6.20 CG: 50.9 ± 5.80	2 weeks, 7 times/week, 90 min/each	G + M	Paper crafts Sticking Origami	HAMD MMSE	*p* < 0.05 *p* < 0.005
24	Wang et al. [46] *	Research article	N = 53 (EG = 25/CG = 28)	24/29	EG: 17.96 ± 3.71 CG: 18.21 ± 2.83	4 weeks, 2 times/week, 90 min/each	G + M	Painting Clay Mandala painting	MCCB SES	** *p* = 0.332 *p* < 0.001
25	Xu et al. [47] *	Research article	N = 70 (EG = 35/CG = 35)	EG: 19/16 CG: 17/18	EG: 23.1 ± 3.60 CG: 23.8 ± 3.90	12 weeks, 1 time/week, 60 min/each	G + M	Painting Collage	SDS Observation of adverse effects	*p* < 0.05 *p* < 0.05
26	Liu and Lu [48] **	Research article	N = 60 (EG = 30/CG = 30)	36/24	All: 45–80	8 weeks, 3 times/week, 60 min/each	G + M	Painting Mandala painting	HAMD HAMA	*p* < 0.01 *p* < 0.01
27	Kang [49] *	Research article	N = 36 (EG = 18/CG = 18)	EG: 10/8 CG: 10/8	EG: 16.75 ± 2.12 CG: 17.73 ± 1.05	24 weeks, NR	G + M	Not recorded	HAMD HAMA	*p* < 0.05 *p* < 0.05
28	Niu et al. [50] *	Research article	N = 60 (EG = 30/CG = 30)	EG: 14/16 CG: 13/17	EG: 34.1 ± 7.80 CG: 34.5 ± 7.10	4 weeks, 5 times/week, 90 min/each	G + M	Painting	HAMD Observation of adverse effects	*p* = 0.04 ** *p* > 0.05
29	Wang [51] *	Research article	N = 36 (EG = 18/CG = 18)	14/22	All: 46.78 ± 11.93	4 weeks, 2 times/week, 90 min/each	G + M	Painting	SDS Self-reported scale of group membership	*p* = 0.002 *p* < 0.001
30	Chen and Yu [52] **	Research article	N = 64 (EG = 32/CG = 32)	23/41	All: 18–56	4 weeks, 1 time/week, 75 min/each	G + M	Origami	HAMD	*p* < 0.01
31	Wang and Di [53] **	Research article	N = 64 (EG = 32/CG = 32)	0/64	All: 48.0 ± 6.0	12 weeks, 7 times/week, 120 min/each	G + M	Knitting	SSPI NOSIE	*p* < 0.01 * *p* < 0.05
32	Wang et al. [54] *	Research article	N = 59 (EG = 30/CG = 29)	EG: 14/16 CG: 13/16	NR	12 weeks, 3 times/week, 120 min/each	G + M	Drawing Painting	SDSS GQOLI-74	*p* < 0.001 * *p* < 0.001
33	Wu et al. [55] **	Research article	N = 60 (EG = 30/CG = 30)	EG: 18/12 CG: 20/10	EG: 49.9 ± 6.30 CG: 49.7 ± 7.50	24 weeks, 7 times/week, 60 min/each	G + M	Knitting Paper crafts Sticking	HAMD BI MMSE	* *p* < 0.05 *p* < 0.05 *p* < 0.005
34	Zheng et al. [22] *	Research article	N = 61 (EG = 31/CG = 30)	EG: 12/19 CG: 10/20	EG: 37.5 ± 12.50 CG: 36.6 ± 12.80	24 weeks, 2–3 times/week, 120 min/each	G + M	Calligraphy	HAMD HAMA	*p* < 0.01 *p* < 0.01

* For the medication + art therapy intervention, *p*-values did not show a significant difference in the outcomes for the change in some factors. ** For the medication + nurse-led art therapy (N; Number, M; Mean, SD; Standard Deviation, EG; experimental group, CG; control group, NR; No report, G; group art therapy, P; personal art therapy, M; medication treatment), *p*-values exhibited no significant difference in the outcomes. BAI; Beck Anxiety Inventory, BDI-21; Beck Depression Inventory-21, BI; Barthel Index, CFQ; Cognitive Fusion Questionnaire, DSRSC; Depression Self-rating Scale for Children, ERQ; Emotion Regulation Questionnaire, FMAS; Fugl-Meyer motor function assessment, FSS; Fatigue Severity Scale, GQOLI-74; Generic Quality of Life Inventory-74, GWB; General Well-Being Schedule, HAMD; Hamilton Depression Scale, HAMA; Hamilton Anxiety Scale, HHI; Herth Hope Index, MBI; Modified Barthel Index, MCCB; MATRICS Consensus Cognitive Battery, MMSE; Minimum Mental State Examination, MoCA; Montreal Cognitive Assessment, NOSIE; Nurses’ Observation Scale for Inpatient Evaluation, PANAS; Positive and Negative Affect Scale, PHCSS; Children’s self-concept Scale, RESE; Regulatory Emotional Self-Efficacy, RSS; Ruminative Responses Scale, SAS; Self-Rating Anxiety Scale, SCARED; The Screen for Child Anxiety Related Emotional Disorders, SDS; Self-Rating Depression Scale, SDSS; Social Disability Screening Schedule, SES; Self-esteem scale; SSPI; Scale of Social function in Psychosis Inpatients, TAS-20; Toronto Alexithymia Scale-20, TESS; Treatment Emergent Symptom Scale, WCST; Wisconsin card sorting test, WHOQOL; The World Health Organization Quality of Life.

**Table 4:** Risk of bias assessment results for the randomized controlled studies.

Category	Risk of Bias	n	%
Random sequence generation	Low	17	63.0
	Uncertain	10	37.0
	High	0	0
Allocation concealment	Low	14	51.9
	Uncertain	13	48.1
	High	0	0
Blinding of participants and personnel	Low	0	0
	Uncertain	0	0
	High	27	100
Blinding of outcome assessment	Low	10	37.0
	Uncertain	17	63.0
	High	0	0
Incomplete outcome data	Low	26	96.3
	Uncertain	0	0
	High	1	3.7
Selective reporting	Low	27	100
	Uncertain	0	0
	High	0	0
Other bias	Low	26	96.3
	Uncertain	0	0
	High	1	3.7

**Table 5:** Risk of bias assessment results for the nonrandomized controlled studies.

Category	Classification	n	%
Selection of participants	Low	7	100
	Uncertain	0	37.0
	High	0	0
Confounding variables	Low	5	71.0
	Uncertain	1	14.3
	High	1	14.3
Measurement of exposure	Low	6	85.7
	Uncertain	0	0
	High	1	14.3
Blinding for outcome assessment	Low	3	42.9
	Uncertain	4	57.1
	High	0	0
Incomplete outcome data	Low	7	100
	Uncertain	0	0
	High	0	0
Selective outcome reporting	Low	7	100
	Uncertain	0	0
	High	0	0

**Table 6:** Trends in art therapy research for Chinese patients with depressive disorder.

Classification (*N*)	Result
Research Features	Research on patients with depression began in 2008 and has been increasing, with most of the results published in academic journals (31 papers, 91.2%)
Subject of mediation	Mainly general depression patients with an average age in their 20 s (13 cases, 38.2%)
Mediation Methods	In most studies, the total number of participants was up to 70 (21 studies, 61.7%), the number of interventions was 6 to 15 (20 studies, 58.8%), the duration of each intervention was 60 to 90 min (23 studies, 67.6%), and group therapy was used (32 studies, 94.1%).
Contents of arbitration	Among various art therapy techniques, many studies utilized painting techniques (23 items, 46.9%).

**Table 7:** Effects of art therapy interventions for Chinese patients with depressive disorder.

Classification (*N*)	Specific Variable (*N*)	Result
Emotional domain (31)	Depression (28), Anxiety (14), Positive and Negative Emotions (4), Inability to express Emotions (1), Emotional regulation (1), Happiness (1)	Nearly all of the studies investigating emotional factors reported positive effects. However, Wu et al. (2011) found no significant changes in factors reflecting depressive mood—specifically body weight, diurnal variation, and sleep disturbances—with all *p* values > 0.05, though significant differences were observed in other factors [55]. Similarly, Ran reported no significant difference in the generalized anxiety factor (*p* > 0.05), while significant differences were identified in all other factors measured [37].
Social do-main (12)	Social functioning (5), mental and so-cial functioning (5), quality of life (3), activities of daily living (2)	Majority of the studies investigating social factors reported positive effects. However, Wang and Di (2012) found no significant difference in personal hygiene (*p* > 0.05), although significant differences were observed in all other factors [53].
Cognitive Domain (10)	Cognitive function (6), rumination (2), mental state (2), cognitive integration (1)	All studies investigating cognitive factors reported positive effects.
Self Domain (9)	Self-esteem (5), self-awareness (2), self-efficacy (1), self-evaluation (1), sense of hope (1)	All studies examining self-related factors reported positive outcomes.
Physical Domain (5)	Adverse reactions (3), fatigue (1), mo-tor function; upper limb (1)	Among studies examining physical indicators, three reported posi-tive effects. However, Niu et al. and Ran et al. found no significant difference in the incidence of adverse reactions compared to the con-trol group (*p* > 0.05) [32,50].

## Data Availability

Not applicable.
